# Study on psychoeducation enhancing results of adherence in patients with schizophrenia (SPERA-S): study protocol for a randomized controlled trial

**DOI:** 10.1186/1745-6215-14-323

**Published:** 2013-10-07

**Authors:** Donatella Rita Petretto, Antonio Preti, Carlo Zuddas, Franco Veltro, Marco Bruno Luigi Rocchi, Davide Sisti, Valentina Martinelli, Mauro Giovanni Carta, Carmelo Masala

**Affiliations:** 1 Department of Education, Psychology, Philosophy - University of Cagliari, Cagliari, Italy; 2 ASL 3, Campobasso, Italy; 3 Department Of Biomolecular Sciences, University of Urbino ‘Carlo Bo’, Urbino, Italy; 4 Pharmacology Unit at the University Hospital of Cagliari, University of Cagliari and AOU Cagliari, Cagliari, Italy; 5 Consultation-Liaison Psychiatric Unit at the University Hospital of Cagliari, University of Cagliari and AOU Cagliari, Cagliari, Italy

**Keywords:** Randomized controlled trial, Schizophrenia, Psychoeducation, Falloon’s method, Adherence to pharmacotherapy, Family, Caregiver

## Abstract

**Background:**

Poor adherence to pharmacotherapy negatively affects the course and the outcome of schizophreniaspectrum psychoses, enhancing the risk of relapse. Falloon and coworkers developed a Psychoeducation Program aimed at improving communication and problem-solving abilities in patients and their families. This study set out to evaluate changes in adherence to pharmacotherapy in patients diagnosed with schizophrenia-spectrum psychoses, by comparing one group exposed to the Falloon Psychoeducation Program (FPP) with another group exposed to family supportive therapy with generic information on the disorders.

**Methods:**

340 patients diagnosed with schizophrenia and related disorders according to standardized criteria from 10 participating units distributed throughout the Italian National Health System (NHS), will be enrolled with 1:1 allocation by the method of blocks of randomized permutations. Patients will be reassessed at 6, 12 and 18 months after start of treatment (duration: 6 months).

The primary objective is to evaluate changes in adherence to pharmacotherapy after psychoeducation. Adherence will be assessed at three-month intervals by measuring blood levels of the primary prescribed drug using high pressure liquid chromatography, and via the Medication Adherence Questionnaire and a modified version of the Adherence Interview. Secondary objectives are changes in the frequency of relapse and readmission, as the main indicator of the course of the disorder.

Enrolled patients will be allocated to the FPP (yes/no) randomly, 1:1, in a procedure controlled by the coordinating unit; codes will be masked until the conclusion of the protocol (or the occurrence of a severe negative event). The raters will be blind to treatment allocation and will be tested for blinding after treatment completion. Intention-to-treat will be applied in considering the primary and secondary outcomes. Multiple imputations will be applied to integrate the missing data. The study started recruitment in February 2013; the total duration of the study is 27 months.

**Discussion:**

If the psychoeducation program proves effective in improving adherence to pharmacotherapy and in reducing relapse and readmissions, its application could be proposed as a standard adjunctive psychosocial treatment within the Italian NHS.

**Trial registration:**

Protocol Registration System of ClinicalTrials.gov NCT01433094; registered on 20 August 2011; first patient was randomized on 12 February 2013.

## Background

Schizophrenia and its related psychoses are severe mental disorders with a high impact in terms of disability and poor quality of life. The clinical course of schizophrenia is typically one of highly recurrent acute episodes with chronic impairment of social, vocational and personal wellbeing [1-3]. Prevalence of schizophrenia in the general population is 0.5 to 1%, with a higher risk and poorer outcome among males than females [4].

The costs for patients, their families and society are huge, and largely generated by the direct cost of care, especially hospitalization [5-7]. Even higher costs arise from lost productivity (unemployment of patients and absence from work by the relatives who care for them), informal care, criminal justice service involvement, and social security expenditure [8,9].

Poor adherence to therapy negatively impacts on the course and the outcome of schizophrenia, enhancing the risk of relapse, hospital admission and readmission, and family burden [10]. Non-adherence to pharmacotherapy in patients diagnosed with schizophrenia is 41 to 50% [11,12] and is predictive of a higher risk of relapse and a readmission rate up to five times higher than in adherent patients [13].

Currently, pharmacotherapy is the most important therapeutic intervention in the treatment of schizophrenia-spectrum psychoses. Higher adherence to pharmacotherapy is expected to favor a better course of the disorder, and specifically to reduce the risk of relapse and readmission, both of which heavily increase the burden of the disorder and are a great cause of distress, particularly after compulsory admission.

In recent years, many educational programs have been aimed at improve knowledge of the disorder, its symptoms, course and outcome, and the availability of treatment, and have focused specifically on the patients and their families [14-17].

Psychoeducational programs were developed to improve adherence to pharmacotherapy by reducing irrational beliefs towards drugs and their side effects, increasing tolerance to the inevitable and unwanted effects, and promoting coping strategies and problem-solving skills to help face everyday problems associated with the disorder. Studies on the real effectiveness of these programs have found protective effects against the risk of relapse and on the probability of readmission, with medium effect-sizes: Cohen’s d = 0.18 (95% CI = 0.13 to 0.49) and d = 0.58 (95% CI = 0.27 to 0.89), respectively [16]. A 2011 meta-analysis including 5,142 participants from 44 independent trials conducted from 1998 to 2009 confirmed the effectiveness of psychoeducation in reducing relapse and readmission [18]. Participants receiving psychoeducation were also found to be more satisfied with mental health services and reported a better quality of life.

More complex intervention models involve both the patients and their families, because the disorder is chronic from its onset and can cause a heavy burden on the family: the patient is unlikely to preserve a reasonable level of autonomy and independence without the support of his/her relatives. For this reason, psychoeducation programs involving the family are more likely to produce positive results on the course of the disorder [16].

Falloon and co-workers were among the first to develop a model of intervention in schizophrenia centered on the psychoeducation of the patients and their families [19,20]. Falloon’s model included sessions aimed at helping patients and their families recognize early stressful events and early indicators of the risk of stress-induced relapse; sessions aimed at the development of optimal adherence to pharmacotherapy; and sessions aimed at improving coping strategies and problem solving in everyday life [20]. This method proved effective following the first evaluation: at the two-year follow-up, cumulative relapse rates markedly differed between controls and the interventional group (83% versus 17%, respectively), and family therapy according to Falloon’s model resulted in fewer hospitalizations, improvement in patients’ social functioning, and lower levels of family burden and distress [19]. Past studies on Falloon’s model did not investigate adherence to pharmacotherapy in detail but concentrated on the course of the disorder and, specifically on the risk of relapse and readmission. Several more studies confirmed the effectiveness of this method on the risk of hospitalization, family burden and patient’s social functioning [20] but did not report clear evidence of effects on adherence, a result expected on the theoretical basis of the model and supported in small-sample studies carried out in specialized academic settings [21]. The results of these latter studies cannot be extended to the General Hospital Psychiatric Units (GHPUs) operating in the Italian National Health System (INHS).

Currently, expert consensus guidelines assign family psychoeducation a high second-line rating and recommend it to address adherence problems in patients with severe mental disorders [22]. In the INHS, family psychoeducation is rarely provided to patients and their families, with the exception of academic settings [23]. There is evidence of an inverse relationship between the availability of community-based mental health care and the need status of schizophrenic patients: the fewer out-patient and rehabilitation services available, the more unmet needs there were [24]. Improving the effectiveness of treatment offered to patients who have been diagnosed with schizophrenia is also likely to reduce the stigma of schizophrenia [25], which is barely affected by the small increase in public understanding of the biological correlates of mental illness [26].

### Aims

This study specifically set out to investigate the effectiveness of Falloon and co-workers’ Psychoeducation Program (FPP) and to evaluate its feasibility as part of the standard care setting provided by the INHS. The FPP will be compared to a control group, and randomized to a treatment with generic information on the disorders administered with the same frequencies as the FPP (Generic Treatment (GT)). We foresee that the FPP will be effective in improving adherence to pharmacotherapy in the exposed group when compared to the non-exposed group, and we also expect that the FPP will reduce the risk of relapse and readmission in the exposed group, by maintaining its effects at both six-and twelve-month follow-ups. Positive results would imply the importance of applying the FPP in all INHS settings, and the need of exploring its potential in other classes of psychosis, such as affective psychoses, which are also burdened by high personal, family and social costs.

A secondary goal of the study is the production of a manual to be used in the training and assessment of the professional staff involved in the treatment of patients diagnosed with schizophrenia-spectrum psychosis.

The primary outcome of the study, adherence to therapy, will be measured through a triple approach: a scaled self-report questionnaire, a four-query interview and, for the first time, the blood levels of the primary prescribed drug (to be measured by a specialized pharmacology unit with extensive experience in this type of studies). In this way, we will be able to validate the simpler instruments of measurement, such as the self-report or interview, with an objective, gold-standard method (blood levels of the therapeutic compound), a practice rarely used in past studies on adherence to therapy in patients with psychosis [27]. If one or both of the simpler, self-report instruments of assessment prove to be valid enough to differentiate adherent from non-adherent or partially adherent patients, the study will produce add-on information on easy-to-be-administered tools, to be generalized for future related studies.

The participating INHS centers are distributed across the country: we foresee the participation of ten enrolling units from nine large administrative regions of north, central and south Italy, to include both GHPUs and University Psychiatric Clinics (UPCs), six of them already having experience with the FPP. The heterogeneity of the participating units can be seen as a limit of the study, when considered in terms of intra-cluster variance (which we took into account in statistical power analysis on sample size, with appropriate corrections). However, it can represent an advantage, too, allowing for the exploration of the feasibility and effectiveness of the FPP in different settings, especially those operating in GHPUs, which have traditionally been exposed to a higher workload than academic centers where the FPP has more often been tested thus far.

## Methods/design

### Overview

This study is supported by the Italian Medicines Agency (Agenzia Italia del Farmaco) - grant for SPERA-S: Study on Psychoeducation Enhancing Results of Adherence in Schizophrenia (FARM892ZXE) (see Acknowledgements for details on the contract of financing).

The institutional review boards of both the university that coordinated the study and of the participating centers approved the study’s protocol which complies with the provisions of the 1995 Declaration of Helsinki (as revised in Tokyo, 2004).

In this study, the FPP will be compared to a treatment providing general information on psychosis GT administered with the same frequency as the FPP. The study will be randomized and blinded for the assessment: raters will be not informed about whether patients are in the FPP or GT groups. Inevitably, the therapists providing the treatment will know their patients’ treatment status.

The study will enroll adult patients diagnosed with psychosis in the spectrum of schizophrenia from ten participating units distributed throughout INHS territory. The sample will be randomized in an exposed group and an unexposed group, with 340 patients overall enrolled, with a 1:1 allocation ratio.

### Eligibility criteria

#### Inclusion criteria

Patients will be enrolled when they have a diagnosis of psychosis in the spectrum of schizophrenia according to the International Classification of Diseases - tenth edition (ICD-10: codes F20 to F29) and have been in contact with the unit for at least 24 months; admitting ages vary from 18 to 55 years old. Enrolled patients will be randomly allocated to FPP treatment (yes/no) with a rate 1:1, via a procedure controlled by the coordinating unit; codes will be masked until the conclusion of the protocol (or the occurrence of a severe negative event). Randomization will be carried out by the method of blocks of randomized permutations (to preserve balancing between the exposed and the non-exposed groups). Randomization will be stratified by location, due to the study’s multisite nature.

#### Exclusion criteria

Exclusion criteria are mental retardation or any severe cognitive impairment; psychosis due to substance abuse or to a medical condition; affective psychosis; co-morbid substance dependence; patient does not understand Italian; pharmacotherapy with depot. Inability or unwillingness to provide informed consent are additional exclusion criteria (see Table 1 for details).

**Table 1:** Eligibility criteria

Inclusion criteria	Diagnosis of psychosis in the spectrum of schizophrenia (ICD-10: F20 to F29)
	Contact with the unit for at least 24 months
	Admitting ages varying from 18 to 55 years
Exclusion criteria	Mental retardation, or any severe cognitive impairment
	Psychosis due to substance abuse or to a medical condition
	Co-morbid substance dependence
	Patient does not understand Italian
	Pharmacotherapy with depot
	Inability or unwillingness to provide informed consent

### Recruitment and baseline procedures

Patients will be enrolled in the clinical setting, which includes both GHPUs and UPCs.

To rationalize the training and quality-control procedures for the FPP and GT therapists (two for each participating unit) and the assessors (one for each unit), a Training and Quality Control Center will be established in Cagliari, the site of the coordination unit. This Center will train the assigned therapists in FPP and GT and will also train the assessors in the administration of the psychometric measures. On a regular six-month basis, the Center will organize meetings to verify the course of the study.

The Coordination Center will manage the procedures of treatment allocation and will coordinate the study, the statistical analysis, the storage of data, the training of the assessors involved in the patients’ evaluation and of the therapists involved in the FPP and GT. The Coordination Center with two units from the University of Cagliari (Center for Research and Treatment in Mental Health and Pharmacological Unit) will monitor data recording and storage, quality assessment of FPP and GT, and quality assessment of the training for assessors and therapists.

A Steering Committee (the principal investigator plus another member of the Coordination Center, a member from the Monitoring Unit, a member from the Statistics Unit, and three members elected by the enrolling units) will coordinate the use of the database for publications. A centralized laboratory (Pharmacological Unit, University of Cagliari) will analyze the blood levels for drugs in the samples received from all participating units.

### Timing

In the first three months of the study, the Coordination Center and all directors of the participating units will check the organization of the study, the assessment methodology, the training of the assessors and their inter-rater agreement.

**Figure 1:**
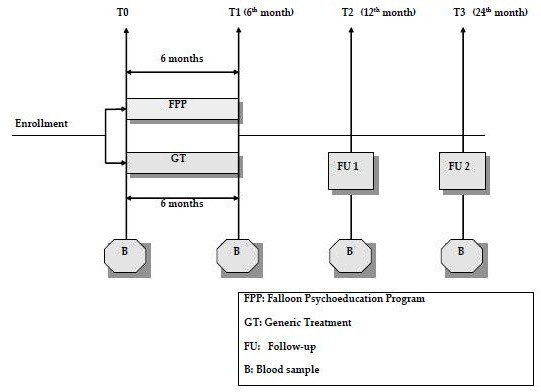
Flowchart of participants during the trial.

As far as the investigational trial is concerned, we foresee three months for the enrollment of the patients; six months for the subsequent exposition to the FPP of the patients allocated to treatment (the exposed group), or same-length GT for the unexposed group; there will be subsequent six-month and twelve-month follow-ups to verify whether the immediate benefit of FPP, if any, is maintained after the conclusion of the FPP treatment. Patients will be evaluated at entry (t0), at the conclusion of the treatment (both exposed and unexposed) (t1), after six months of follow-up (t2) and after twelve months of follow-up (t3). The final report will be made within six months of the conclusion of the study, but initial reports will be prepared six months after the start (see Figure 1).

### Randomization procedures and bias-minimization methods

All patients will be randomized in the exposed (FPP) or the unexposed (GT) group with a 1:1 allocation ratio in each unit, as each unit is expected to participate in the enrollment of both groups. Randomization will be carried out by the method of blocks of randomized permutations (to preserve balancing between the exposed and the non-exposed groups). Randomization will be stratified by location due to the multisite nature of the study. Correctness of the randomization procedure will be monitored at regular intervals.

### Allocation concealment

Block size will be specified by the Coordination Center and will not be revealed to any researcher or staff until the end of the enrollment period.

### Blinding

This study will implement a complete separation of treatment and assessment to allow for a single blind design of assessment. Therefore, therapists will not be involved in the assessment of the treatment outcome, and raters will not be allowed to hold treatment sessions. Patients will be informed about their treatment allocation by the therapist, and only therapists will receive information about group allocation. Discussions about patients enrolled in the study will be not allowed between raters and therapists, as per instructions given during staff training.

The raters will have to complete a ‘blindness protocol’ at the end of the study. Any unintentional disclosure of the treatment condition will therefore be hopefully documented. Further, the raters will be asked to guess the study condition of the patient after the end of the study. Among all documented guesses, the rate of correct guesses should not significantly differ from chance (that is, 50%). The main outcome of the study is adherence to the prescribed therapy, and one of the indicators is the measurement of blood levels of the primary prescribed drug, which is independent from the assessment carried out by the raters. It is unlikely that any personal opinion of the raters on the presumed superior effectiveness of the FPP over GT will influence the measurement of blood levels of the primary prescribed drug. However, we are interested in knowing whether any bias might have occurred in the blinding, whether by unintentional disclosure of the treatment condition or because the personal opinion of the raters led them to guess the patients’ allocation to the treatment modality.

### Planned interventions

The FPP aims at improving communication and problem-solving abilities in patients and their families through sessions focused on: assessment of the individual’s and the family’s strengths, weaknesses, and goals; education about schizophrenia and treatment; communication skills training; problem-solving training; and training to cope with special problems [19]. Treatment sessions are provided on a weekly basis for six months (one and a half hours for each session). The first six sessions will be provided to each individual family with the participation of the patient and the caregivers, while the following sessions up to the 18th will be provided to clusters of families, according to a multi-family revised version of family psychoeducation [28,29].

The GT is a treatment providing general information on the disorders and with the same frequencies as the FPP (Table 2). The GT treatment sessions are also provided on a weekly basis for six months (one and a half hours for each session). Each GT session is structured in three steps: first, a short (20 minutes maximum) and hopefully informative introduction on selected topics concerning the main problems related to the disorder (see Table 2 for details); then family members are asked to discuss whether and how they faced the problem described, whether the solution was effective and why, and they are then are invited to imagine alternative solutions to the problem that differ from the solutions they adopted; finally, a ten-minute conclusion summarizes the main elements of the topic discussed during the meeting. This scheme is specifically effective in multi-family meetings, when different families are invited to discuss and compare the problems they faced and the solutions they adopted, and whether effective or not.

**Table 2:** Timing and structure of the planned interventions: Falloon and co-workers’ Psychoeducation Program (FPP) and Generic Treatment (GT)

**Sequence**	**Month**	**FPP**	**GT**	**Type of meeting**
1 week	1	Introduction	Introduction on schizophrenia	Single family
2 week	1	Evaluation of individual family members	Meeting on delusions, hallucinations and apathy	Single family
3 week	1	Whole family evaluation	Meeting on depression, shame and risk of suicide	Single family
4 week	1	1. Informative meeting on schizophrenia	Meeting on drugs	Single family
5 week	2	2. Meeting on drugs	Meeting on aggressive behavior	Single family
6 week	2	3. Meeting on early signs of relapse	Meeting on psycho-social treatments	Single family
7 week	2	Practical problem solving 1	Meeting on free time, leisure and school	Multi-family
8 week	2	Practical problem solving 2	Meeting on participation in family life	Multi-family
9 week	3	Practical problem solving 3	Meeting on early signs of relapse	Multi-family
10 week	3	Interpersonal problem solving 1	Meeting on eating habits and obsessions	Multi-family
11 week	3	Interpersonal problem solving 2	Meeting on therapy acceptance	Multi-family
12 week	3	Interpersonal problem solving 3	Meeting on self-care and somatic health	Multi-family
13 week	4	Personal problem solving 1	Meeting on disability	Multi-family
14 week	4	Personal problem solving 2	Meeting on stigma	Multi-family
15 week	4	Personal problem solving 3	Meeting on legal problems and substance abuse	Multi-family
16 week	4	First verification of family changes	Meeting on social anxiety	Multi-family
First week of the month	5	Second verification of family changes	Meeting on social services	Multi-family
First week of the month	6	Third verification of family changes	Summary meeting and verification of family changes	Multi-family

Both treatments share some common, non-specific effects, while they differ on their specific effects. The basic strategy in family psychoeducation attempts to reduce the impact of environmental stress on biologically vulnerable individuals by promoting communication within the family, increasing coping skills and improving problem-solving abilities [30]. These outcomes are expected to be produced by: a) providing information about the disorder, which should also decrease the pathogenic impact of ‘false myths’ concerning the illness; b) the provision of coping strategies to manage crises; c) the implementation of a more comfortable environment where support is provided by peers and professionals. Within this framework, non-specific effects include emotional support, empathic listening, and the implementation of therapeutic optimism. Specific effects, expected to influence therapeutic outcome, are thought to be the consequence of specific and well-defined treatment strategies. In the FPP, the specific effects are thought to be the consequences of improved problem-solving, better coping and tolerance to stress, and improved social skills acquired by the intervention. In the GT, the specific effects are essentially the reduction of shame and sense of guilt obtained by explicitly talking about situational problems related to the disorder, and the modelization on the non-judgmental, empathic and supportive behavior of the therapist.

For both the FPP and GT, participation in the meetings by each family member who has expressed willingness to participate will be recorded. A family will be considered adherent to the psychoeducation treatment when at least one member of the family has participated in 70% of the meetings (n = 12 out of 18). The rate of family attendance will be considered as a confounding variable in the evaluation of outcome. Percentage of drop-out by type of psychoeducation treatment (families who have not participated in three successive meetings and have not attended at least 70% of meetings overall) will be considered as an independent measure of applicability of the method (FPP or GT).

### Assessment and outcome measures

Assessment is aimed at investigating the clinical and functional status of the patients and evaluating the main effects of treatment on the primary and secondary outcome measures (Table 3). The primary outcome is the patient’s adherence to pharmacotherapy, intended as the continuous use of the primary prescribed drug. Even if patients with psychosis are generally under poly-pharmacotherapy, only adherence to the most important drug (the one expected to produce the main therapeutic effect), as indicated by the enrolling unit, will be monitored during the study. Adherence will be checked by a triple method of assessment: patient’s self-report on the Medication Adherence Rating Scale (MARS), patient’s replies to the four-query interview of the Brief Adherence Rating Scale (BARS), and measurement of blood levels of the prescribed drug. Data will be analyzed using two indicators of adherence:

1. a dichotomic index, where non-adherence is the lack of adherence to the treatment in 30% or more of the monthly evaluation on the self-report or the interview (since replies on both measures might not coincide);

2. a continuous index based on the scale - self-report or interview - which could be the most reliable by comparison with the measurement of blood levels of the primary prescribed drug, to apply statistical analyses of covariance and verify the impact of confounding variables on the primary outcome.

**Table 3:** Timing of assessments of endpoints for efficacy and safety

	**T0**	**T1**	**T2**	**T3**
Background information (age, sex, diagnosis, and so on)	X			
Clinical Global Impressions	X	X	X	X
Brief Psychiatric Rating Scale (BPRS)	X	X	X	X
Positive And Negative Syndrome Scale (PANSS)	X	X	X	X
Social Performance Scale	X	X	X	X
Health of the Nation Outcome Scales (HoNOS)	X	X	X	X
World Health Organization Quality Of Life (WHOQoL)	X	X	X	X
The Family Questionnaire	X	X	X	X
Medication Adherence Rating Scale	X	X	X	X
Brief Adherence Rating Scale	X	X	X	X
Dosage Record and Treatment Emergent Symptoms Scale (DOTES)	X	X	X	X
Sheenan-Suicidality Tracking Scale (S-STS)	X	X	X	X
Modified Overt Aggression Scale (MOAS) 1.0	X	X	X	X
Blood sample for assessing of the blood level of the prescribed drug	X	X	X	X
Assessment of blinding				X

A number of related, secondary outcomes will be assessed: the occurrence of psychotic symptoms, according to the Brief Psychiatric Rating Scale (BPRS) and the Positive and Negative Syndrome Scale (PANSS); the general level of psychopathology, according to the Health of the Nation Outcome Scales (HoNOS); changes in social functioning, according to the Personal and Social Performance scale (PSP), and in the quality of life, as evaluated on the WHO-Quality of Life-Short form (WHOQOL-Bref); changes in the patient’s overall clinical status, as measured by the Clinical Global Impression (CGI); changes in the dosage of the prescribed drugs, as a reflection of changes in adherence to therapy; and, finally, changes in the frequency of relapse and readmission. Italian validated versions of the scales will be used. The BARS and the Family Questionnaire (see below) were translated for this study.

### Assessment tools

The MARS is a ten-item yes/no self-report instrument. Total scores vary from 0 (low likelihood of medication adherence) to 10 (high likelihood). From this scale, we used the five items specifically referring to the taking or not of the medication [31,32]. Therefore in our MARS version, total scores vary from 0 (low likelihood of medication adherence) to 5 (high likelihood).

The BARS is a brief, pencil-paper, clinician-administered adherence assessment instrument [33]. The BARS consists of four items: three questions and an overall visual analog rating scale to assess the percentage of doses taken by the patient in the past month (0% to 100%). The visual analog scale rating serves as a final adherence determination. The three questions inquire about patients’ knowledge of their own medication regimen and episodes of missed medication taking, and include: number of prescribed doses per day (question 1); number of days, over the past month, the patient did not take the prescribed doses (question 2); and number of days, over the past month, the patient took less than the prescribed doses (question 3). A higher score on the BARS corresponds to better adherence.

The CGI is a tripartite rating scale aimed at assessing the severity of the patient at the moment of the assessment, improvement or worsening relative to the baseline, and efficacy of treatment (described in [34]). The CGI-severity rating is provided on a seven-point scale that assesses severity of the patient on the basis of total clinical impression: 1) not at all ill; 2) borderline mentally ill; 3) mildly ill; 4) moderately ill; 5) markedly ill; 6) severely ill; or 7) extremely ill. The CGI-change is provided on a seven-point scale, rated as: 1) very much improved; 2) much improved; 3) minimally improved; 4) no change; 5) minimally worse; 6) much worse; or 7) very much worse. The CGI-efficacy index is a rating scale crossing the efficacy of the treatment by occurrence of side effects: 1) unchanged to worse; 2) minimal efficacy; 3) moderate efficacy; 4) marked efficacy, with side effects rated as: 1) none; 2) does not interfere with patient’s functioning; 3) interferes with patient’s functioning; 4) outweighs therapeutic effects. A higher score on the CGI-severity and the CGI-change rating corresponds to greater severity and worsening, respectively. Conversely, a higher score on the CGI-efficacy index corresponds to better efficacy (however, greater side effects counterbalance efficacy).

The BPRS is a 24-item measure of general psychopathology in a Likert format, with scores from 1 (absent) to 7 (extremely severe) [35,36]. Possible BPRS total scores varied from 24 to 168. The primary purpose of the BPRS is to allow assessment of treatment change across a comprehensive set of common symptom characteristics. The original 16-item BPRS was initially expanded to 18 items, then to 24 items by adding 6 symptom items (bizarre behavior, self-neglect, suicidality, elevated mood, distractibility, and motor hyperactivity) to increase sensitivity to a broader range of psychotic and affective symptoms [37]. A higher score on the BPRS corresponds to greater psychopathology.

The PANSS is a measure of the current symptoms of patients on a 30-item scale [38,39]. The PANSS consists of three domains: positive symptoms, negative symptoms, and general psychopathology. Items are rated from 1 (absent) to 7 (extremely severe); the total score varies from 30 to 210. A higher score on the PANSS corresponds to greater psychopathology.

The HoNOS comprises 12 items that rate various aspects of mental and social health with a severity score varying from 0 (no problem) to 4 (severe to very severe problem). These items are grouped in four subscales: behavioral problems, impairment problems, symptoms problems and social problems [40]. Clinicians rate HoNOS before and after interventions, so that changes attributable to the interventions (outcomes) can be measured [41]. A higher score on each HoNOS subscale corresponds to greater occurrence of problems in the corresponding areas.

The PSP is a version of the Global Assessment of Functioning [42], with detailed instructions on how to rate the functioning of the patient [43]. The PSP rates the social and occupational functions of patients; its scores vary from 1 to 100, with a score of 100 indicating excellent functioning.

The WHOQOL-Bref is a relatively new instrument, used to measure quality of life. It is an abbreviated version of the WHOQOL-100 quality of life instrument, developed by the WHOQOL group [44,45]. The WHOQOL-Bref adopts the following definition of health-related quality of life: ‘the value assigned to duration of life as modified by the impairments, functional states, perceptions, and social opportunities that are influenced by disease, injury, treatment, or policy’ [46]. It produces scores for four domains related to the quality of life (physical health, psychological sphere, social relationships and the environment). The Italian version used in this study was also reported to have satisfactory psychometric properties [47]. The items are rated on a five-point Likert scale, reflecting intensity, capacity, frequency or evaluation. The items inquire ‘how much’, ‘how completely’, ‘how often’, ‘how good’ or ‘how satisfied’, with possible answers varying from ‘very satisfied’ to ‘not at all satisfied’. The scores in each domain vary from 4 to 20, where a higher score indicates a better quality of life.

### Expressed emotion in the patient’s family

The concept of expressed emotion (EE) was developed to describe the emotional environment and the attitude of caregivers towards a relative affected by a disorder. The Camberwell Family Interview (CFI) is the standard reference for this type of study [48], and measures the amount of critical comments (CCs), hostility (H), or emotional over-involvement (EOI) expressed by a close relative when talking about a mentally or physically ill family member. High EE consistently predicts the risk of relapse for patients with schizophrenia [49,50], but the CFI is time-consuming, and requires detailed training of the assessors. The Family Questionnaire (FQ) is a brief self-report questionnaire aimed at measuring expressed emotion on a four-point Likert scale, from 0 (never/rarely) to 4 (often/always) [51]. The FQ provides two subscale scores: critical comments (CC) and emotional over-involvement (EOI). Scores of 24 or higher on the CC subscale or 28 or higher on the EOI subscale define the occurrence of EE in the family. The FQ was proved to relate to EE rating by the CFI. FQ scores will be analyzed both as a confounding variable to explore the role of EE in the effectiveness of psychoeducation, and as an outcome measure to evaluate whether FPP or GT can improve EE levels in the patient’s family.

### Remission

According to the Remission in Schizophrenia Working Group, remission for patients with schizophrenia is defined as a mild score (= 3 or lower) over a six-month period on all eight items of PANSS considered representative of the core symptoms of psychosis: delusions, hallucinations, positive formal thought disorder, bizarre behavior, affective flattening, avolition-apathy, anhedonia-asociality, alogia; or a mild score (= 3 or lower) over a six-month period on all seven items of BPRS considered representative of the core symptoms of psychosis: grandiosity, suspiciousness, unusual thought content, hallucinatory behavior, conceptual disorganization, mannerism/posturing, blunted affect [52,53].

### Relapse

For the purpose of this study, relapse is defined as any score equal to five or higher - for one month out of two or more - on the core symptoms of psychosis on the BPRS or PANSS, as defined above.

### Readmission rate

Readmission is defined as any admission to a psychiatric unit to treat symptoms of psychosis or to change the drug regimen. Admissions for somatic reasons will not be considered as episodes of readmission. All voluntary and involuntary admissions of participants at enrollment during the study and at follow-up will be recorded.

### Suicidal ideation and suicidal behavior

For the purpose of this study, suicide attempt is defined as any act of purposeful self-harm with expressed suicidal intent, that is, the wish to die, as reported by the patient at the assessment or by a family member consulted as a key informant (no discrepancy), or recorded during treatment, within the first year of follow-up. Suicidal ideation will be measured with the Sheehan Suicidality Tracking Scale (S-STS). The S-STS is an eight-item prospective rating scale that tracks treatment-emergent suicidal ideation and behaviors [54,55]. Each item in the S-STS is scored on a five-point Likert scale (0 = not at all, 1 = a little, 2 = moderately, 3 = very, and 4 = extremely). Data from the S-STS can be analyzed as individual item scores, a suicidal ideation subscale score (sum of scores from items 2, 3, and 4, plus score from item 5 if ≥ 1), a suicidal behavior subscale score (sum of scores from items 6, 7a, and 8, plus score from item 5 if > 1), and as a total score. A higher score on the S-STS and its subscales corresponds to greater risk of suicide.

### Aggressive behavior

Aggressive behavior will be monitored with the Modified Overt Aggression Scale (MOAS). The MOAS rates overt aggression displayed by the patient in the period preceding the assessment [56,57]. Ratings are provided for the most severe act in four categories: verbal aggression, aggression against objects, aggression against oneself, and aggression against other people. Aggressive acts are rated from 0 (no aggressive behavior) to 4, with increasing severity from 1 to 4; then, scores are multiplied by a factor specific for each category: 1 for verbal aggression, 2 for aggression against objects, 3 for aggression against oneself, and 4 for aggression against other people [58]. The total score varies from 0 (no aggression) to 40 (maximum grade of aggression). A higher score on the MOAS and its subscales corresponds to a higher occurrence of aggressive behaviors.

### Side effects

Side effects to drugs will be closely monitored and coded with the Dosage Record and Treatment Emergent Symptoms Scale (DOTES). The DOTES is a 30-item scale aimed at recording changes in the patient’s health status that can or could be attributed to the prescribed psychopharmacological treatment (described in [34]). The scale lists symptoms and pathological conditions affecting the patient’s body, with a judgment required on their presence and severity, and a separate assessment of the actual or potential links with the patient’s prescribed drugs. The presence and severity subscale includes 4 levels, from 1 (absent) to 4 (severe, resulting in drug suspension). The subscale on the actual or potential links with the patient’s prescribed drugs includes 5 levels, from 1 (symptom absent or no link), to 5 (certain link, as certified in the literature). A higher score on the DOTES corresponds to a higher occurrence and severity of side effects, which may or may not be related to the patient’s prescribed drugs on the basis of the rating of the subscale on the actual or potential links.

### Follow-up assessment

The FPP has a six-month duration, so patients will be assessed at entry (t0), at the end of the FPP (t1), at the six-month follow-up (t2), and at the twelve-month follow-up (t3), for secondary outcome measures. Adherence will be monitored at t0, t1, t2, and t3, via self-reports, interviews and measured blood levels of the primary prescribed drug.

### Sample size and power

Sample size for the identification of the expected difference in the primary outcome (adherence to pharmacotherapy) was based on known evidence: prevalence of non-adherence to pharmacotherapy in patients already in contact with a psychiatric service is around 40 to 50%, median = 47% [11,12]; effect size of psychosocial treatment on various outcomes, including relapse, readmission and adherence to pharmacotherapy is 0.48 of the standard deviation (SD), with 95% CI= 0.10 to 0.85 [16]. We expect that our intervention could produce a change in the prevalence of non-adherence to pharmacotherapy in the exposed group with an effect size of 0.45 SD [11,12]. After performing a test of difference between proportions we determined that a sample of 78 participants per group would be needed to achieve 80% power to detect a difference with h = 0.45 (medium effect size) in the measures of adherence to pharmacotherapy, at a two-sided significance level of 0.05 and allocation ratio of 1:1.

Cohen’s h effect size for the difference of proportions is calculated on the formula:

$$h=2^{*}\arcsin\;p1-2^{*}\arcsin\;p2$$

where p1 and p2 are the proportions of adherent patients in the exposed group 1 and the non-exposed group 2, respectively [59]. The Cohen’s h measures the same effect size interval as the Cohen’s d but is based on differences by proportion rather than by continuous variables [59].

Cohen’s h = 0.45 implies that at the estimated power (= 80%) we can observe a 25.5% rate of non-adherence to pharmacotherapy in the exposed group as opposed to a 47% rate in the unexposed group. In this case, risk reduction is 0.215, corresponding to a number needed to treat (NNT) = 5. Since we do not know the exact proportion of non-adherence, here are the different starting p1 and related maximum p2 detectable:

p1: 0.65 0.60 0.55 0.50 0.45 0.40 0.35 0.30 0.25

p2: 0.43 0.38 0.33 0.28 0.24 0.20 0.16 0.12 0.09

Since this a multisite study, the clusterized nature of the data has to be controlled since it has an impact on an unequal variance by center, which requires a correction of the sample according to the formula:

$$\mathrm{DE}=1+\left(m-1\right)\times r$$

where m is the original sample size per cluster (in our study, m = 7.8 + 7.8 = 15.6 about 16), r is a value summarizing the intra-cluster correlation coefficient (ICC), and DE (design effect) is the correction needed to maintain the same effect size in the expected change produced by the treatment [60]. In most studies, the ICC varies between 0.02 and 0.05. With ten enrolling units, precautionary ICC equals 0.05 and DE 1 + (16–1)*0.05 = 1.75. Therefore, by multiplying the initial sample size (n = 156) by DE, the final ICC-corrected sample size is 273, equal to 27.3 about 28 patients per cluster.

### Medication

The study is open about the psychopharmacological treatment, with no treatment restrictions. On the basis of the study’s sample size, we expect equal distribution of type (classical versus atypical antipsychotics), dose, rate of non-adherence to medication, and prescription of other psychopharmacological treatment (antidepressants, mood stabilizers, benzodiazepines) in both treatment groups. Since the type and medication dose are potential confounders, they will be controlled.

### Data management and analysis

All analyses will be based, as far as possible, on the intention-to-treat principle. Differences in the primary outcome (adherence to pharmacotherapy) will be analyzed with a two-tail test for proportions with the Miettinen exact test [61]. A multiple logistic regression will be carried out to control for the confounding variables [62] using adherence (yes/no) as the outcome to verify the impact of the FPP on controlling the impact of the confounding variables (sex, age, severity of the disorder, cluster).

Survival analyses will be performed using the Kaplan-Meier method, followed by the log-rank test to define the terminal events at both the start of non-adherence and the first relapse or readmission episode [63]. Intention-to-treat will be applied considering the primary and secondary outcomes [64]. Multiple imputations will be applied to integrate missing data [65].

As far as validation of the self-report (MARS) and the interview (BARS) measure of adherence with respect to the blood levels of the main prescribed drug is concerned, Spearman’s rho will be used to compare blood levels of the main drug with the self-report or the interview; partial correlation will be used to ascertain the links between blood levels of the main drug with the self-report or the interview by taking into account the other measures (the interview or the self-report, respectively).

All analyses will be done with Excel (Microsoft Excel 2013 running on Windows 8) (Chicago, IL 60606, USA) or the Statistical Package for Social Science (SPSS) version 17 for Windows.

### Ethical considerations

The FPP is not expected to produce relevant somatic side effects but it can produce unwanted or unforeseen effects, such as an increase in the intra-family level of conflict. To maximize safety for the patients, before randomization each patient will be evaluated on his or her risk of unwanted or unforeseen effects attributable to exposure to the FPP.

The therapists involved in the administration of the FPP and GT will be monitored to minimize any improper method of administration of the protocol of treatment; assessors will also be trained and monitored to avoid dispersion of information, thus violating the guaranteed criterion of good faith in case of patients’ disclosing sensitive information. Data recording and storage will comply with the privacy law in force, and informed consent for information recording will be obtained from each patient.

Participation in the study is voluntary and written informed consent is obtained. Before enrollment, participants will receive both verbal and written assurance that they can withdraw from the trial any time, with no consequence for their continued treatment. Patients unable to give informed consent in an appropriate way or who refuse to participate in the study will not be enrolled.

The Training Center is committed to monitoring the quality of assessment and of the administration of the FPP procedures, on a three-month basis.

A paradoxical, unintended effect of greater adherence to pharmacotherapy is the potential increase of the somatic side effects of the prescribed drugs. Indeed, there is some indirect evidence that this can actually occur [66]. To limit this paradoxical effect, patients will be closely monitored for side effects and somatic conditions on a three-month basis or as needed. It is expected that, following improvement in the response to the therapy as a result of greater adherence to pharmacotherapy, the therapist will decrease dosage of the prescribed drugs. This specific change will be checked at assessment and a reduction of dosage of the prescribed drugs and of poly-pharmacotherapy is among the secondary outcomes of the study.

Regarding the obligation to professional secrecy, this is the responsibility of the enrolling unit, and all data are recorded anonymously and coded with a concealed script, which will not be disclosed until the end of the study. Only the Coordination Center owns these concealed codes, not to be disclosed to the statistical units, which will receive the data with a neutral assignment (group A, group B).

### Safety

Safety will be closely monitored. Key endpoints will be:

•death caused by suicide

•suicide attempt

•suicidal crisis (explicit plan for serious suicidal activity without suicide attempt)

•severe symptomatic exacerbation, defined by the Clinical Global Impression Scale (CGI), which includes ratings of illness severity and changes in overall clinical status. A rating of CGI-severity ≥ 6 (severely ill) and CGI-change ≥ 3 (much worse) would be regarded as severe symptomatic exacerbation.

Information about these safety variables is recorded in the Case Report Form (CRF) as part of the regular clinical assessment.

## Discussion

To the best of the authors’ knowledge, this is the first study in Italy concerning the implementation of a protocol of family psychoeducation within the framework of the National Health System. Positive findings, if any, might be extended and generalized to the whole National Health System, which is an advantage over past studies. Indeed, past studies principally involved specialized academic settings [21,67], or included small samples in very specific settings [68]. In the past, limited benefit was reported in the routine clinical practice ([27], even though a different view was expressed by Rummel-Kluge and Kissling [17]).

Another advantage of this study over past investigations is its sample size, which is planned to be large enough to allow multivariable analyses to discover specific factors involved in the effectiveness of psychoeducation, and to control for confounding factors and covariates. Finally, the study checks adherence to therapy as the main outcome measure using both self-report and interview on the one hand, and blood levels of the main prescribed drug on the other hand. This will prevent a biased measurement of adherence to therapy which can occur with subjective measures like self-report and interviews.

Another important point is the use of a control group based on a different approach to psychoeducation. This will allow for the evaluation of the most specific effects of the FPP, its effectiveness and specificity. We cannot exclude that the GT approach could result as effective as the FPP in improving adherence to treatment and in decreasing relapse and readmission over time. Since we do not have a third study group on standard care because of budget limits, we will be unable to establish whether the changes produced by FPP or GT are really superior to the changes that can be produced by standard care over time. Equal effectiveness also points towards superiority of the less expensive and time-consuming method - the GT - which might be preferred over the FPP since its implementation requires less therapist training. A proper economic analysis is necessary to assess superiority of one method over the other in case of equal effectiveness.

### Potential limitations

The study protocol imposes a detailed assessment of symptoms and patient’s functioning, and patients’ willingness to be assessed in such a detailed manner could be limited. Calculation of sample size included a 20% drop-out rate. Because of the complexity of the assessment protocol, dropouts might be more than 20%, thus hampering the extent of the multivariable analyses on the whole sample. Patients could also refuse blood sampling and not permit the main gold standard of adherence measurement.

## Trial status

The study started recruiting participants in February 2013, and the recruitment is ongoing.

## Abbreviations

BARS: Brief adherence rating scale; BPRS: Brief psychiatric rating scale; CCs: Critical comments; CFI: Camberwell family interview; CGI: Clinical global impression; CGI: Clinical global impression scale; CRF: Case report form; DE: Design effect; DOTES: Dosage record and treatment emergent symptoms scale; EE: Expressed emotion; EOI: Emotional over-involvement; FPP: Psychoeducation program falloon; FQ: Family questionnaire; GHPUs: General hospital psychiatric units; GT: Generic treatment; H: Hostility; HoNOS: Health of the nation outcome scales; ICC: Intra-cluster correlation coefficient; INHS: Italian national health system; MARS: Medication adherence rating scale; MOAS: Modified overt aggression scale; NNT: Number needed to treat; PANSS: Positive and negative syndrome scale; PSP: Personal and social performance scale; SD: Standard deviation; S-STS: Sheehan suicidality tracking scale; UPCs: University psychiatric clinics; WHO-QOL-Bref: WHO-quality of life-short form.

## Competing interests

The authors declare that they have no competing interests.

## Authors’ contributions

DRP is the principal investigator and grant holder of the SPERA-S study and is - together with CM - speaker of the SPERA-S research network. MGC wrote the first draft of the project and organized the network of the collaborating centers. DRP, AP, FV, MBLR, DS, VM, MGC and CM all contributed to the design of the study. CZ contributed to the protocol with qualitative data analysis. DRP, AP, CM, MBLR, DS and FV drafted the paper. All the authors have revised the paper and have approved the final manuscript.
